# PACT/RAX Regulates the Migration of Cerebellar Granule Neurons in the Developing Cerebellum

**DOI:** 10.1038/srep07961

**Published:** 2015-01-22

**Authors:** Yue Yong, Ya Meng, Hanqing Ding, Zhiqin Fan, Yifen Tang, Chenghua Zhou, Jia Luo, Zun-Ji Ke

**Affiliations:** 1 Department of Biochemistry, Shanghai University of Traditional Chinese Medicine, 1200 Cailun Road, Shanghai 201203, China; 2 Department of Pharmacology and Nutritonal Sciences, University of Kentucky College of Medicine, Lexington, Kentucky 40536, U.S.A; 3 Key Laboratory of Nutrition and Metabolism, Institute for Nutritional Sciences, Shanghai Institutes for Biological Sciences, Chinese Academy of Sciences, Graduate School of the Chinese Academy of Sciences, Shanghai 200031, China

## Abstract

PACT and its murine ortholog RAX were originally identified as a protein activator for the dsRNA-dependent, interferon-inducible protein kinase PKR. Recent studies indicated that RAX played a role in embryogenesis and neuronal development. In this study, we investigated the expression of RAX during the postnatal development of the mouse cerebellum and its role in the migration of cerebellar granule neurons (CGNs). High expression of RAX was observed in the cerebellum from postnatal day (PD) 4 to PD9, a period when the CGNs migrate from the external granule layer (EGL) to the internal granule layer (IGL). The migration of the EGL progenitor cells *in vivo* was inhibited by RAX knockdown on PD4. This finding was confirmed by *in vitro* studies showing that RAX knockdown impaired the migration of CGNs in cerebellar microexplants. PACT/RAX-regulated migration required its third motif and was independent of PKR. PACT/RAX interacted with focal adhesion kinase (FAK) and PACT/RAX knockdown disturbed the FAK phosphorylation in CGNs. These findings demonstrated a *novel* function of PACT/RAX in the regulation of neuronal migration.

Protein kinase, interferon-inducible double stranded RNA dependent activator (PACT) and its murine ortholog RAX were independently discovered as the protein activator for the double strand RNA (dsRNA)-dependent, interferon-inducible protein kinase (PKR)^1^,^2^. PACT and RAX are almost identical in their amino acid sequences and they belong to an evolutionarily conserved family of RNA-binding proteins^3^. Under various stress conditions^4^,^5^,^6^,^7^,^8^, PACT/RAX binds to PKR through its two dsRNA binding motifs (dsRBMs), and regulates the conformational change of PKR through its third motif, resulting in PKR autophosphorylation^9^ and then the phosphorylation of eukaryotic initiation factor 2α (eIF2α), leading to the inhibition of protein synthesis and the induction of apoptosis^10^. PACT also interacts with Dicer to facilitate the maturing process of small RNAs^11^,^12^. The depletion of PACT affects the accumulation of mature microRNAs (miRNAs) and reduces the efficiency of small interfering RNA (siRNA)-induced RNA interference (RNAi)^13^.

The ablation of the 8^th^ exon in the *prkra* gene in mice induces severe microtia, impaired hearing, reduced body size and fertility defects^14^,^15^. Missense mutation in the second dsRBM of the *prkra* gene causes deficits in growth, ear development, craniofacial development and ovarian structure^16^. In addition, deletion of the entire RAX gene is embryonic lethal in mice at the pre-implantation stage. In fruit flies, a transposon insertion in the 5′-UTR of dRax (independently identified as loqs/R3D1) induces a highly abnormal commissural axon structure of the central nervous system (CNS) and 70% of the flies homozygous for the mutant allele die prior to adulthood^17^. All these findings suggest that PACT/RAX plays an important role in embryogenesis and development.

Focal adhesion kinase (FAK) is a tyrosine kinase localizing at the focal adhesions^18^. The regulatory role of FAK or paxillin in cell migration has been well established^18^,^19^. In neurons, phosphorylation of FAK at serine 732 is critical for the organization of a small network of microtubules that partially encompass the nucleus, which is important for neuronal migration^20^. Mice with neuron/glia-specific FAK ablation show impaired cerebellar foliation, such as variable decreases in foliation sizes and the lack of intercrural and precentral fissures^21^.

In this study, we show that the expression of RAX in the cerebellum is developmentally regulated. RAX knockdown impairs cerebellar granule neuron (CGN) migration. The third conserved motif of PACT/RAX is required for its role in migration which is independent of PKR and may be mediated by its interaction with FAK. These results reveal a *novel* role of PACT/RAX in regulating neuronal migration during the development.

## Results

### Developmental expression of RAX in mouse cerebellum

**Figure 1:**
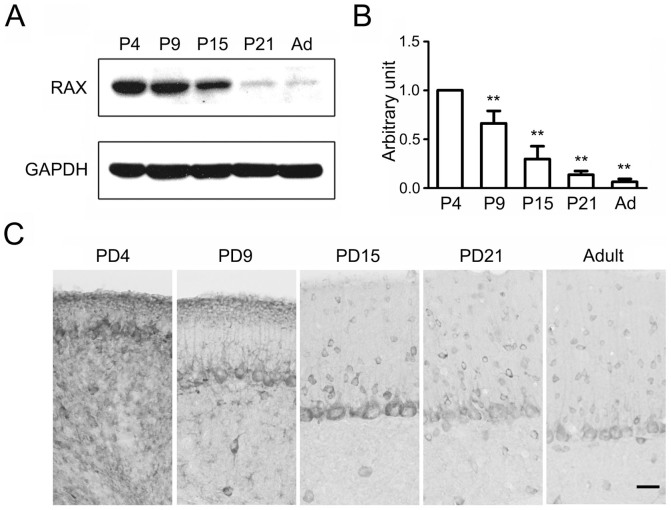
RAX expression in developing mouse cerebellum. (A) The expression of RAX protein in mouse cerebellum at PD4, PD9, PD15, PD21 and adult was measured by immunoblotting. The cropped lines are used and full-length immunoblots are shown in Supplementary Information section (Supplementary Figure 3A). (B) The expression of RAX was quantified and normalized to the loading control GAPDH. Each data point was mean ± s.d. (n = 3), **p < 0.01. (C) The expression of RAX in the developing and adult mouse cerebellum was examined by immunohistochemistry (IHC). Scale bar = 20 μm.

To explore the role of PACT/RAX in cerebellar development, we first examined the developmental expression of RAX in mouse cerebellum. High level of RAX was observed in the cerebellum on PD4 and PD9; the expression decreased thereafter (Figure 1A). Compared to PD4, the expression of RAX decreased 70%, 86% and 94% by PD15, PD21, and adult, respectively (Figure 1B). The immunohistochemical (IHC) staining showed that RAX was highly expressed in EGL and Purkinje cell layer (PL) on PD4 and PD9 (Figure 1C), but the RAX-positive cells were only observed in Purkinje cells and interneurons in the internal granule layer (IGL) and molecular layer (ML) at PD15, PD21 and adulthood (Figure 1C). Confocal microscope images showed that RAX was expresseed in almost all cells in the EGL of PD4 mouse cerebellum (Supplementary Figure 1).

### Role of RAX in the migration of cerebellar granule neurons (CGNs) *in vivo*

**Figure 2:**
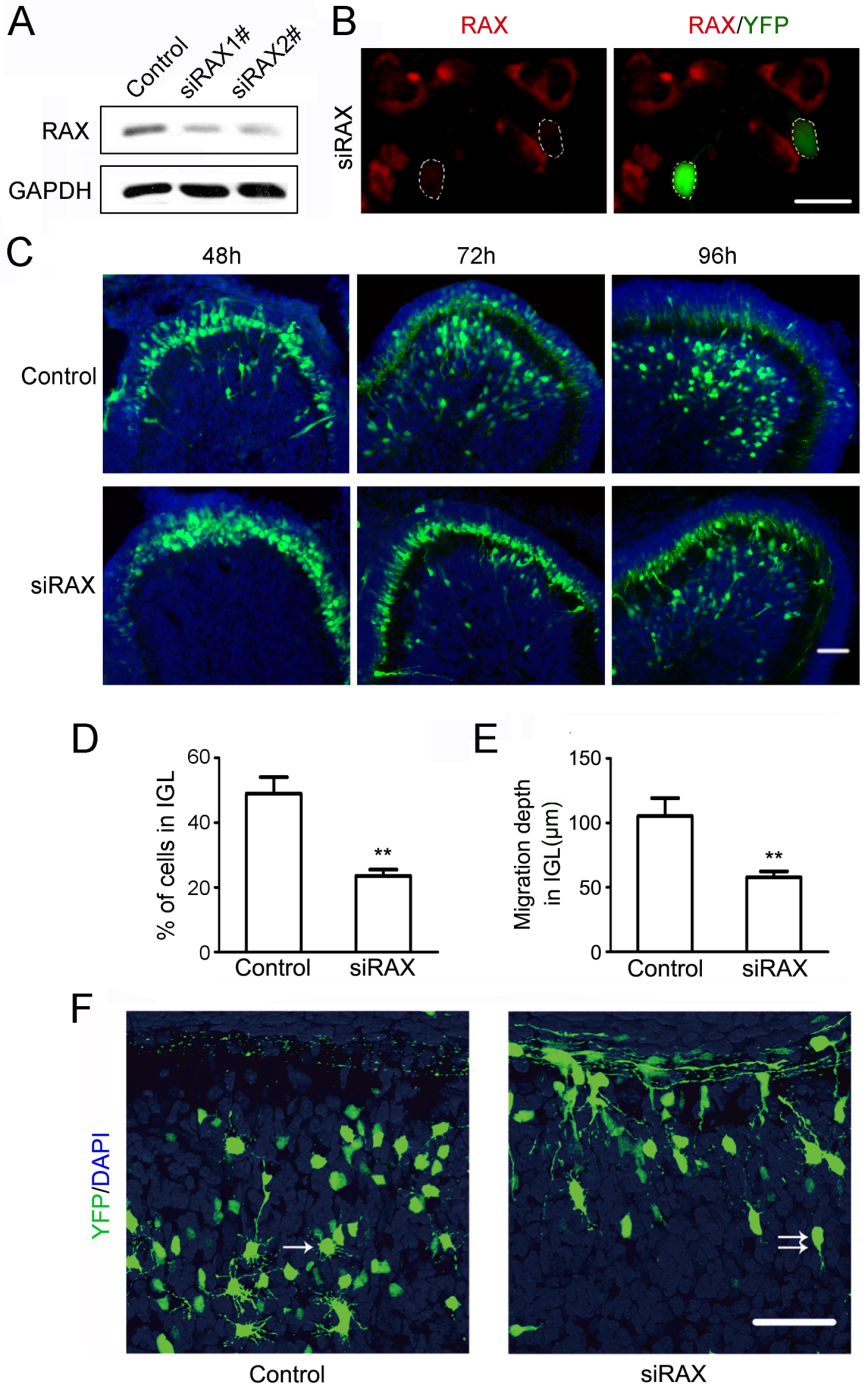
Effect of RAX knockdown on the migration of cerebellar granule neurons (CGNs). (A) Down-regulation of RAX after transfection of RAX siRNA (#1 and #2) for 48 hours was verified by immunoblotting in primary cultured CGNs. The cropped lines are used and full-length immunoblots are shown in Supplementary Information section (Supplementary Figure 3B). (B) Immunofluorescence images showing the RAX (red) expression in CGNs transfected with RAX siRNA for 48 hours (green) or untransfected. Scale bar = 20 μm. (C) siRNA for RAX and control siRNA were delivered to the cerebellum by *in vivo* electroporation at PD4. At 48, 72 or 96 hours post the electroporation, the mice were sacrificed and the sagittal sections of cerebella were examined under a fluorescent microscope as described under the Materials and Methods. Scale bar = 50 μm. (D) The percentage of cells that migrated into IGL was calculated at 72 hours post the electroporation. (E) The depth of cell migration into IGL at 72 hours post the electroporation. Each data point was mean ± s.d. (n ≥ 5), **p < 0.01. (F) Coronal sections of cerebella showing CGNs in control and RAX siRNA (siRAX) treated groups 96 hours post the electroporation. CGNs in the IGL displayed post-migratory morphology of multi-polar processes (arrows in control group). Migrating CGNs showed tangential or radial migratory morphology of bipolar processes (double arrows in siRAX group). Scale bar = 50 μm.

To explore the function of RAX in the developing cerebellum, the small interfering RNA (siRNA) constructs targeting RAX were delivered to the cerebellar granule precursors in the EGL of PD4 mice by *in vivo* electroporation. Enhanced yellow fluorescent protein (EYFP) was used as a marker of gene delivery. The RAX siRNA effectively down-regulated the expression of RAX in primary cerebellar granule neurons (CGNs) as determined by immunoblotting and immunofluorescence (Figures 2A and B). At 48 h post electroporation, some EYFP positive cells were observed migrating from EGL to IGL in the group transfected with a control siRNA; while few EYFP positive cells is migrating in the group transfected with RAX siRNA (Figure 2C). In the control group, 49% of the EYFP-positive cells migrated from outer EGL through inner EGL, and traveled through the ML to reach the IGL within 72 hours following the electroporation. However, only 23% of EYFP-positive cells migrated into IGL (Figures 2C and D) in mice treated with RAX siRNA; the migration from the outer EGL to the inner EGL seemed not affected by RAX knockdown (Figures 2C and D). Also, the migration depth of CGNs was greatly reduced in RAX knockdown mice as compared to control mice; it decreased from 105 μm in control group to 58 μm in RAX knockdown group at 72 hours after the electroporation (Figure 2E). At 96 hours after the electroporation, more EYFP-positive cells migrated to the IGL; but still fewer migrated cells were observed in mice treated with RAX siRNA (Figure 2C). The EYFP-positive cells in the IGL of control mice showed a typical post-migration morphology with multipolar dendrites (Figure 2F, arrow), while the EYFP-positive cells had a migrating bipolar morphology in the RAX knockdown mice (Figure 2F, double arrows) 96 hours post the electroporation. At 7 days post electroporation, 93% of the EYFP-positive cells migrated into IGL in the control group, while only 77% of EYFP-positive cells were in IGL in the siRAX group (Supplementary Figure 2).

**Figure 3:**
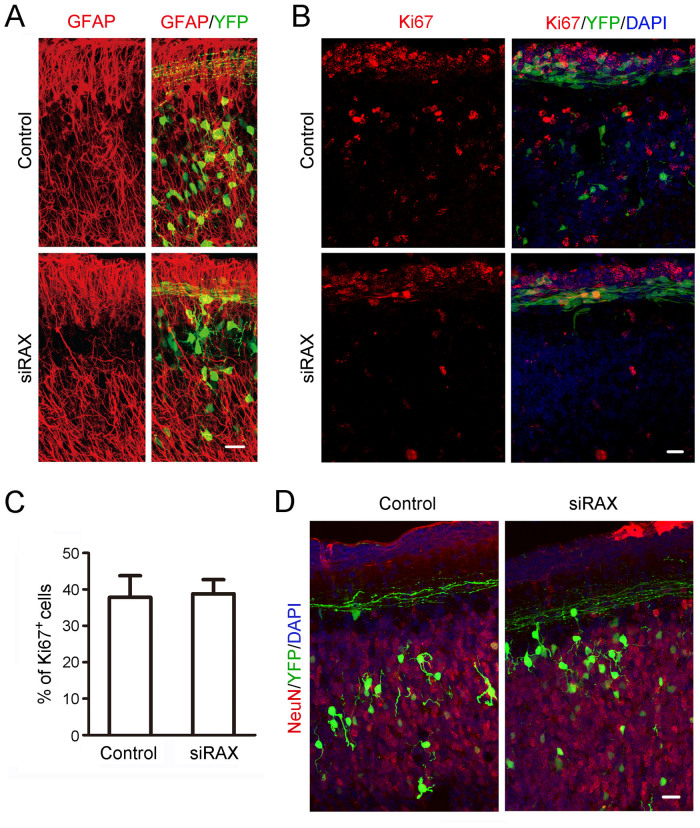
Effect of RAX knockdown on Bergman glia morphology and CGNs proliferation/differentiation. (A) Immunofluorescent images showing Bergman glia and their fibers which were visualized by GFAP staining. Scale bar = 25 μm. (B) At 48 hours post electroporation, sagittal sections of cerebella from both control and siRAX-treated groups were labeled with ki67 antibody (red) and DAPI (blue). Scale bar = 25 μm. (C) The percentage of ki67 positive cells in total transfected cells was calculated. (D) At 7 days post electroporation, coronal sections of cerebella from both control and siRAX-treated groups were labeled with NeuN antibody (red) and DAPI (blue). Scale bar = 25 μm.

Bergman glia is the migration scaffold for CGNs and displayed no morphological difference in both control and RAX knockdown groups as revealed by GFAP staining (Figure 3A). RAX knockdown did not change cell proliferation since the percentage of ki67 positive cells showed no difference between the control siRNA- and RAX siRNA-treated groups (Figures 3B and C). At 7 days post electroporation, the EYFP-positive CGNs migrated into IGL in both groups differentiated and expressed NeuN (Figure 3D). We also examined the expression of cleaved caspase-3 and show no difference between control and RAX knockdown groups (data not shown). Taken together, these results suggested that RAX knockdown inhibited the migration of CGNs without affecting the Bergman glia fibers, CGN proliferation/survival and post-migration differentiation.

### RAX knockdown inhibits the migration of CGNs in cerebellar microexplants

**Figure 4:**
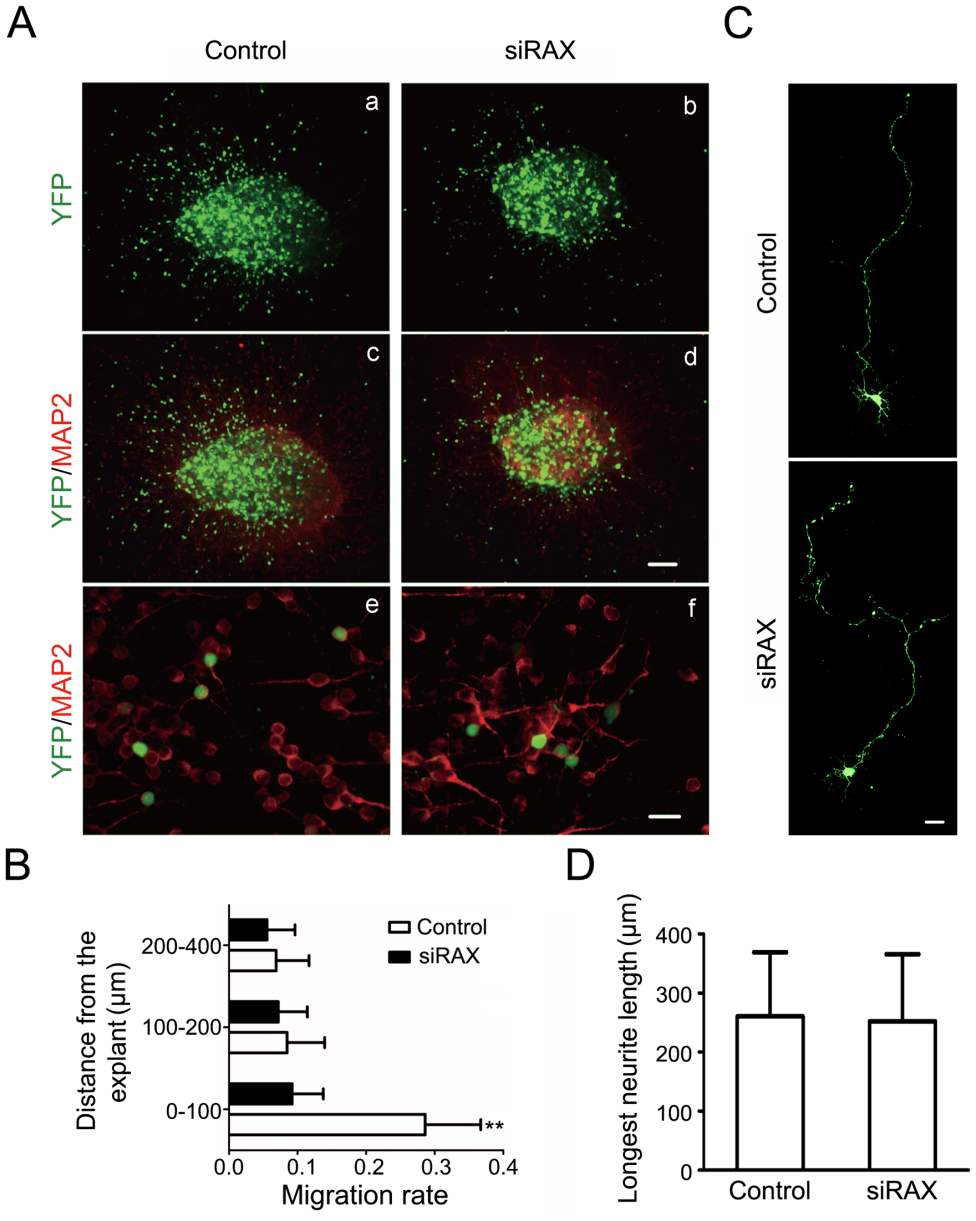
Effect of RAX knockdown on CGN migration in cerebellar microexplants. (A) Cerebellar microexplants were prepared from PD6 mice that were transfected with siRNA for RAX and control siRNA by electroporation at PD4. MAP2 immunofluorescence (IF) was used to visualize neurons migrated out from microexplants after being cultured for 36 hours. YFP-positive signal indicated the transfected cells. Scale bars = 100 μm (a, b, c and d) and 20 μm (e and f). (B) The CGNs migrated out of microexplants were measured by the intensity of YFP fluorescence at various distances (0–100; 100–200 and 200–400 ìm), the intensity of YFP fluorescence within each area was counted and then expressed as a percentage of YFP intensity of the whole. Each data point is mean ± s.d., (n = 10), **p < 0.01. (C) Images of higher magnification showing the migrated neurons. Scale bar = 20 ìm. (D) The length of the longest neurite was quantified in 50 neurons. Each data point is mean ± s.d. from three replicated experiments.

We further examined the role of RAX in CGN migration by using the cultured cerebellar microexplants from PD6 mice. These mice were transfected with control or siRNA for RAX on PD4. We demonstrated that the migration of CGNs was significantly reduced in RAX knockdown group without apparent morphological alterations. The percentage of neurons that migrated within 100 μm was significantly reduced in RAX knockdown group (Figures 4A and B). Neurite growth was not affected by RAX knockdown (Figures 4C and D). These results supported that RAX played an important role in regulating the migration of CGNs.

### The third motif of RAX is essential for its role in regulating CGN migration

**Figure 5:**
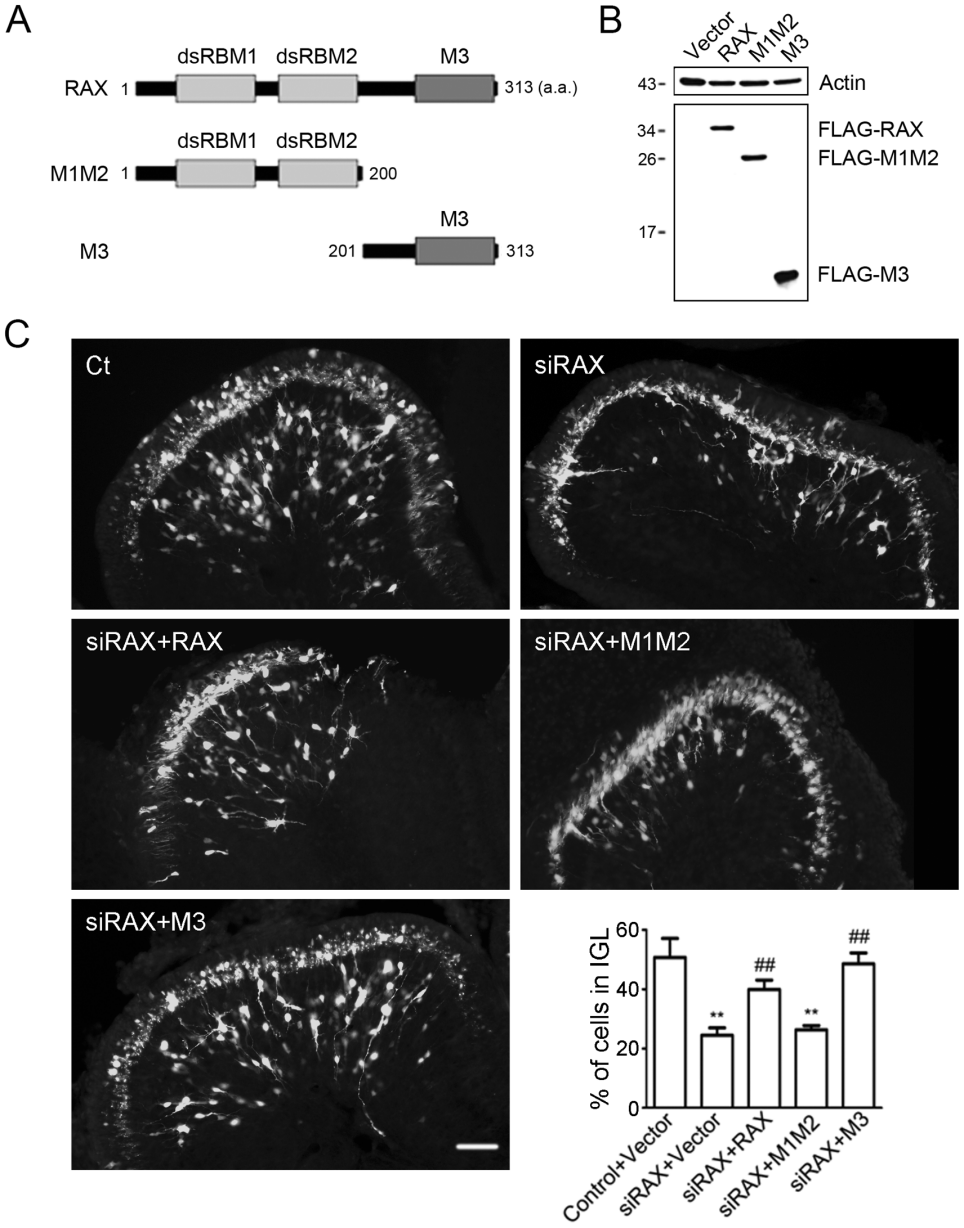
The third motif of RAX was required for the migration of CGNs in the developing cerebellum. (A) Schematic illustration of the wild type and truncated constructs (RAX, M1M2 and M3) of RAX. M1M2 construct contains two dsRBMs. M3 contains the third conserved motif. (B) The expression of truncated RAX in COS7 cells was measured by immunoblotting targeting Flag-tag. The cropped lines are used and full-length immunoblots are shown in Supplementary Information section (Supplementary Figure 3C). (C) Mouse pups were transfected with indicated RAX constructs (RAX, M1M2 and M3) or empty vectors (Vector) and siRAX by electroporation at PD4. Pups were sacrificed 72 hours after the electroporation, and cerebellar sagittal sections were prepared for the examination of cell migration. Scale bar = 50 μm. The percentage of cells that migrated into the IGL was calculated at 72 hours post electroporation. Each data point is mean ± s.d. (n = 5). ** p < 0.01, compared with Control + Vector group, ## p < 0.01, compared with siRAX + Vector group.

RAX is a dsRNA binding protein and has three highly conserved motifs. We generated three recombinant RAX constructs. The first one was a wild type (WT) RAX with synonymous mutation in our siRNA targeting sequence to avoid silencing by the RAX-siRNA. The second one was truncated M1M2 (1–200 aa) including first two dsRBMs with the same mutation; the third one was truncated M3 (201–313 aa) including the third motif (Figure 5A). The expression of these constructs was detected by the expression of the Flag-tag (Figure 5B). When co-transfected with RAX siRNA, both the WT RAX and M3 constructs alleviated RAX knockdown-induced impairment of CGN migration in the developing cerebellum; however, the M1M2 construct failed to reverse the impairment of CGN migration (Figure 5C). These data indicated that the third motif of PACT/RAX was essential for regulating cell migration.

### RAX regulation of CGN migration is PKR-independent

**Figure 6:**
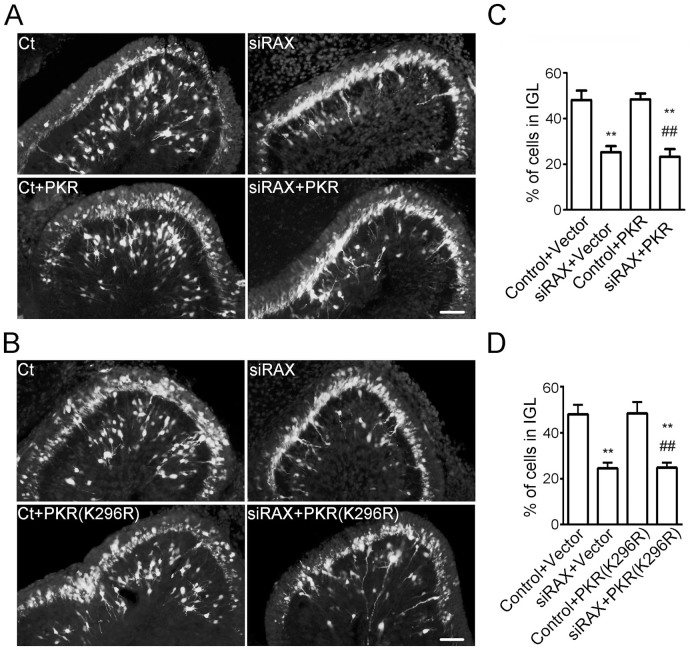
RAX knockdown inhibited CGN migration independent of PKR *in vivo*. (A) and (B) Mouse pups were co-transfected for indicated constructs [siRAX, PKR, PKR (K296R)] by electroporation at PD4, and sacrificed 72 hours after the electroporation. The cerebellar sagittal sections were prepared. YFP positive cells indicated transfected cells. Scale bar = 50 μm. (C) The percentage of YFP-positive granule cells migrated into the IGL (A) was calculated at 72 hours after the electroporation. Each data point is mean ± s.d. (n = 5). ** p < 0.01, compared with Control + Vector group, ## p < 0.01, compared with Control + PKR group. (D) The percentage of YFP-positive granule cells migrated into the IGL (B) was calculated at 72 hours after the electroporation. Each data point is mean ± s.d. (n = 5). ** p < 0.01, compared with Control + Vector group, ## p < 0.01, compared with Control + PKR(K296R) group.

PACT/RAX is the protein activator of PKR and the activation is mediated by the third motif of PACT/RAX^9^,^22^. We therefore sought to determine whether RAX regulation of neuronal migration depended on PKR. Up-regulation of PKR did not alleviate RAX knockdown-induced impairment of CGN migration in the developing cerebellum (Figures 6A and C). Furthermore, transfection of a dominant-negative PKR (K296R) construct did not aggravate the deficit in migration caused by RAX knockdown (Figures 6B and D). These results indicated that RAX regulation of CGN migration was independent of PKR.

### PACT interacts with FAK and is involved in FAK phosphorylation in CGNs

**Figure 7:**
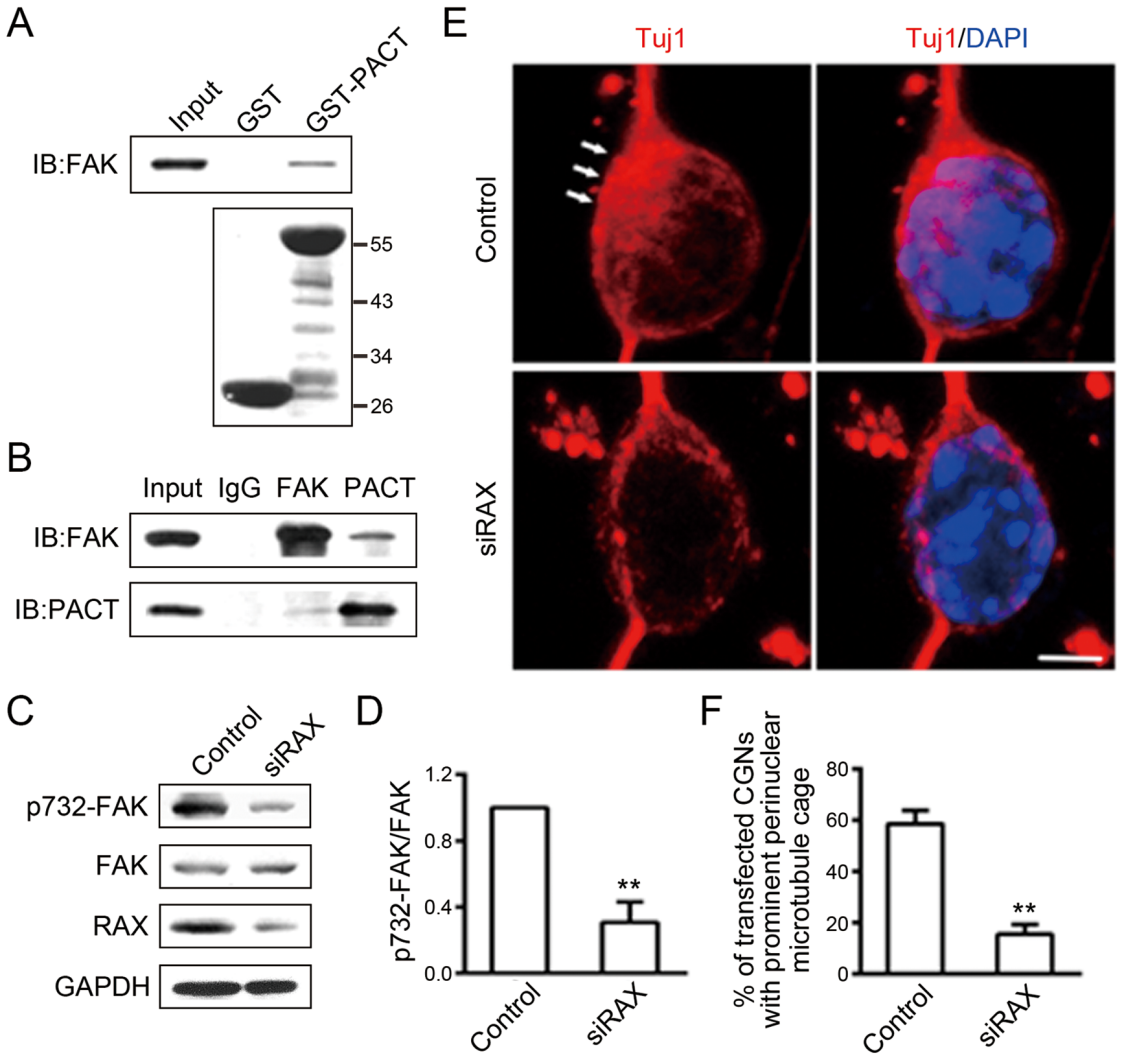
Interaction between PACT/RAX and FAK. (A) PACT and FAK association was determined by GST pull-down assay *in vitro* using COS7 cell lysate. The cropped lines are used and full-length immunoblots are shown in Supplementary Information section (Supplementary Figure 3D). (B) Endogenous PACT and FAK interaction was determined by co-IP in COS7 cells. The cropped lines are used and full-length immunoblots are shown in Supplementary Information section (Supplementary Figure 3E). (C) CGNs were transfected with control siRNA or siRAX for 36 h and then the phosphorylation of FAK at serine 732 (S732) was determined by immunoblotting. The cropped lines are used and full-length immunoblots are shown in Supplementary Information section (Supplementary Figure 3F). (D) Relative amount of pS732-FAK (C) was quantified. Each data point is mean ± s.d.. The experiment was replicated three times. ** p < 0.01. (E) CGNs which were treated with control siRNA or siRAX for 36 hours were double labeled with Tuj1 (red) and DAPI (blue). Scale bar = 5 μm. (F) Quantification of CGNs with prominent perinuclear microtubule cage in control siRNA and siRAX-treated groups (E). Each measurement was based on at last 50 CGNs. Each data point is mean ± s.d.. The experiment was replicated three times. ** p < 0.01. The gels have been run under the same experimental conditions.

FAK is an important regulator of cell migration^18^,^19^. In neurons, phosphorylation of FAK at serine 732 is critical for the organization of a small network of microtubules which is important for neural migration^20^. We found PACT interacted with FAK by GST pull-down assay (Figure 7A) which was also confirmed by co-immunoprecipitation (Figure 7B). In addition, RAX knockdown significantly decreased the phosphorylation of FAK at serine 732 in primary cultured CGNs (Figures 7C and D). Furthermore, RAX knockdown disrupted the organization of microtubules (Figure 7E); 58% of CGNs in the control group displayed prominent perinuclear microtubule cage while only 15% of CGNs transfected with RAX siRNA had this structure (Figure 7F).

## Discussion

The human protein PACT and its mouse ortholog RAX were discovered independently as the cellular activator for the interferon-inducible, dsRNA-dependent protein kinase PKR^1^,^2^. It has been reported that female flies homozygous for the *dRax* deletion are sterile^23^, and display defects in CNS development^17^. The deletion of exon 8 of mouse *Rax* causes developmental defects, including reduced size and severe microtia^14^. The loss of the entire RAX gene in mice induced an early developmental lethality at a pre-implantation stage^17^. These results indicate that RAX plays a role in the embryogenesis and development. In this study, we demonstrate that RAX plays an important role in the development of mouse cerebellum.

High levels of RAX in mouse cerebellum at PD4 and PD9 are consistent with the time period in which CGNs migrate from the EGL to the IGL. Immunohistochemical staining shows that RAX is highly expressed in EGL and Purkinje cells layer, suggesting a role in the migration of CGNs (Figure 1). We demonstrate that RAX knockdown causes granule cells to accumulate at the inner border of the EGL where the granule cell migration turns from tangential to radial, and RAX knockdown also affects the distance which granule cells migrate within the IGL (Figure 2). However, RAX knockdown does not damage the migration scaffold, the radial fibers of Bergmann glia; it does not affect either the proliferation/survival or differentiation of CGNs (Figure 3). It is therefore likely RAX regulates the ability of neuronal migration. This conclusion is supported by an *in vitro* study showing that RAX knockdown blocks neuronal migration in cultured cerebellar microexplants (Figure 4).

A recent study shows that PKR is involved in the migration of breast cancer cells; the activation of PKR suppresses cell motility by regulating the p38 MAPK/MK2/LIMK/cofilin pathway^24^. Our results indicate that up-regulation of PKR expression could not rescue RAX knockdown-induced inhibition of CGN migration and co-expression of dominant negative PKR (K296R) does not aggravate the migration defect caused by RAX knockdown (Figure 6). Therefore, PACT/RAX regulation of CGN migration is likely independent of PKR.

PACT/RAX contains three independent motifs. The first two resemble dsRBM; the third one consisting of 66 residues appears to be an activator for interacting proteins^22^. The dsRBMs of PACT (motif 1 and 2) interact with the dsRBMs in PKR; but the ability to activate PKR is imparted by the third motif of PACT that binds to the PKR kinase domain^9^. Binding of motif 3 to PKR causes PKR auto-phosphorylation by converting PKR from inactive conformation to an active one^25^. Although PACT's third motif binds weakly to PKR, it is necessary and sufficient to activate PKR^9^. Motif 1 and 2, on the other hand, may facilitate the process by mediating strong interaction between PACT and PKR. In mammalian cells, PACT itself is phosphorylated during various chemical stresses or growth factor withdrawal, and the phosphorylation may induce conformational changes of PACT to facilitate its third motif to associate with PKR^8^,^26^,^27^. We demonstrate that the expression of recombinant third motif of RAX is sufficient to reverse the defects in migration caused by RAX knockdown in the developing cerebellum. On the other hand, the expression of other two motifs fails to rescue CGNs from RAX knockdown-induced inhibition of neuronal migration (Figure 5). These results indicate that the third motif of PACT/RAX is critical for its regulation of neuronal migration. Interestingly, it appears that PKR is not involved in this process. Over expression of WT PKR does not alleviate the deficiency of CGN migration caused by RAX knockdown; dominant negative PKR (K296R PKR) is unable to aggravate the deficiency (Figure 6). PKR is not the only protein that interacts with the 3rd domain of PACT/RAX. Other well-known dsRBPs, such as Dicer and trans-activation responsive RNA-binding protein (TRBP), also interact with this domain. There may be other unknown factors that could interact with this domain. Currently, it is unclear how the third motif regulates CGN migration and what its interacting proteins are.

FAK plays an essential role in the regulation of cell migration^18^,^19^. By co-IP and GST pull-down assays, we demonstrate the interaction between PACT/RAX and FAK. The phosphorylation of FAK at serine 732 is critical for organization of a small network of microtubules that partially encompass the nucleus which is important for neuronal migration^20^,^28^. We show that knockdown of PACT/RAX disturbs phosphorylation of FAK (S732) in CGNs (Figure 7). This suggests that PACT/RAX is upstream of FAK which is a critical mediator of neuronal migration.

Hence, our study unravels RAX is a new regulator of CGN migration during cerebellum development. Deficiency of RAX induces CGN migration defects, which is PKR independent, but the third motif of RAX is functionally necessary. Furthermore, knockdown of RAX decreases FAK serine 732 phosphorylation and disrupts the organization of microtubules. This study provides a novel insight into the regulatory mechanism of CGN migration and the potential involvement of RAX in this process.

## Methods

### Reagents and animals

All culture dishes and plates were obtained from Corning Inc. (Corning, NY), and the culture coverslips were from Glaswarenfabrik Karl Hecht GmbH & Co KG (Sondheim/Rhön Germany). Laminin, Poly-D-Lysine, and 3′3-diaminobenzidine (DAB) were obtained from Sigma-Aldrich Co. LLC. (St. Louis, MO). Protein A/G PLUS-Agarose was obtained from Santa Cruz Biotechnology, Inc. (Santa Cruz, CA). Anti-cleaved-Caspase 3 antibody was obtained from Cell Signaling Technology, Inc. (Beverly, MA). Anti-PACT and anti-FAK were obtained from Santa Cruz Biotechnology, Inc. (Santa Cruz, CA). Anti-GFAP antibody was obtained from Sigma-Aldrich Co. LLC. (St. Louis, MO). Anti-Tuj1 antibody was obtained from Covance (Emeryville, CA). Anti-NeuN antibody was obtained from Millipore (Billerica, MA). Anti-ki67antibody was obtained from Abcam (Cambridge, UK). Anti-GAPDH antibody was obtained from Kangcheng Bio-tech Inc. (Shanghai, China). Anti-pS732-FAK antibody and Alexa Fluor® 555 conjugated phalloidin were obtained from Invitrogen Inc. (Grand Island, NY). Alexa-labeled secondary antibodies were obtained from Molecular Probes (Eugene, Oregon).

All experimental protocols were approved by Institute for Nutritional Sciences, Shanghai Institutes for Biological Sciences (SIBS), Chinese Academy of Sciences (CAS). C57BL/6J mice were obtained from Shanghai SLAC Laboratory Animal Co. Ltd (Shanghai, China). The procedure for animal surgery was performed in accordance with the Guideline of Animal Care and Use Committee of the Institute for Nutritional Sciences. Every effort was made to minimize the number of animals used and their suffering.

### Plasmids construction and cell transfection

In order to reduce the RAX mRNA level, small hairpin RNAs (shRNA) targeting the RAX sequence were generated using BLOCK-iT™ RNAi designer (Invitrogen Inc., Grand Island, NY). The oligonucleotides specific for the RAX gene were as follows:

1# sense primer: 5′-CACCGGACCTTCAGTTTGGGCAAGACGAATCTTGCCCAAACTGAAGGTCC-3′, antisense primer: 5′-AAAAGGACCTTCAGTTTGGGCAAGATTCGTCTTGCCCAAACTGAAGGTCC-3′; 2# sense primer: 5′-CACCGCAAGATGATAACAGCTAAGCCGAAGCTTAGCTGTTATCATCTTGC-3′, antisense primer: 5′-AAAAGCAAGATGATAACAGCTAAGCTTCGGCTTAGCTGTTATCATCTTGC-3′;

3# sense primer: 5′-CACCGGAATTAGCAATTCACCATGGCGAACCATGGTGAATTGCTAATTCC-3′, antisense primer: 5′-AAAAGGAATTAGCAATTCACCATGGTTCGCCATGGTGAATTGCTAATTCC -3′. The sense and antisense primers were annealed to generate double-stranded oligonucleotides that were ligated into the pENTR/U6 vector (Invitrogen Inc., Grand Island, NY). A vector containing LacZ targeting sequence was used as a control. DNA constructs were transfected into primary cultured cerebellar granule neurons (CGNs) with electroporation by Amaxa™ Nucleofector™ Technology.

Mouse recombinant RAX with flag-tag was cloned into pCAGGS-IRES-EGFP vector using the primers as follows: FP 5′-CCGCTCGAGCTCGCCATGTCCCATAGC-3′, RP 5′-TCCCCCCGGGCTACTTATCGTCGTCATCCTTGTAATCCTTTCTTTCTGCTATTATCTTTAAATAC-3′. Then the synonymous mutation (from ^61^TTCAGTTTG^69^ to ^61^TTTTCACTG^69^) was generated using KOD -Plus- Mutagenesis Kit (TOYOBO)to avoid silencing by the shRNA plasmid 1# using the primers as follows: FP: 5′-GGACAGCGGGACCTTTTCACTGGGCAAGATGATAAC-3′, RP: 5′-GTTATCATCTTGCCCAGTGAAAAGGTCCCGCTGTCC-3′. The truncations of RAX were constructed with the mutated template using the primers as follows: M1M2: FP: 5′-CCGCTCGAGCTCGCCATGTCCCATAGC-3′, RP: 5′-TCCCCCCGGGCTACTTATCGTCGTCATCCTTGTAATCGTGGTTCTCTGGAGAAATATTACTAAAC-3′; M3: FP: 5′-CCGCTCGAGATGATTTCTCTAACGAACGTGGTTGG-3′, RP: 5′-TCCCCCCGGGCTACTTATCGTCGTCATCCTTGTAATCCTTTCTTTCTGCTATTATCTTTAAATAC-3′.

Human recombinant GST-PACT fusion protein was cloned into p-GEX-6p-2 vector using the primers as follows: FP 5′-CGGGATCCATGTCCCATAGCAGGCATCG-3′, RP 5′-CCGCTCGAGCTACTTTCTTTCTGCTATTATCTTTAAATACT-3′.

Human wild type PKR and dominant negative PKR (K296R) constructs were a kind gift from Dr. Alan Hinnebusch (National Institutes of Health, Bethesda, Maryland). All plasmids were verified by DNA sequencing.

### *In vivo* gene delivery and quantification of CGN migration

Plasmids were delivered to the cerebellum by *in vivo* electroporation as described previously with some modifications^29^,^30^. Briefly, C57BL/6J mouse pups of postnatal day (PD) 4 were anesthetized, and the interparieta bone was exposed and pierced. Plasmids in sterile water containing 0.01% fast green was injected onto the surface of the cerebellum cortex. For each pup, 1.5 μl of plasmid (3 μg/μl) was injected. Electroporation (4 pulses of 100 V for 50 microseconds with 950 microseconds intervals) was carried out using an Electro Square Porator (ECM 830, BTX). These pups were sacrificed 1, 2, 4, or 7 days following electroporation and the cerebella were harvested.

Five slices with highest transfection efficiency from each mouse were selected for quantifying the migration of CGNs. The lobules 4/5 or 6 were examined. We evaluated the percentage of cells that migrated into the IGL as well as the distance that they migrated inside the IGL (depth of migration). The depth of migration was determined by the distance the YFP-positive cell traveled within the IGL.

### Culture and analysis of CGNs and cerebellar microexplants

Mouse pups that received *in vivo* gene deliveries on PD4 were sacrificed 48 hours following the electroporation, and the external granule layer (EGL) containing YFP positive cells was dissected under a dissecting microscope. Some tissues were prepared for primary CGN culture and others were used for cerebellar microexplant culture.

Primary CGN cultures were established as previously described^31^. Briefly, the dissected tissues were dissociated by trypsin incubation and trituration, and then centrifuged. The cell pellet was re-suspended in Neurobasal medium containing B27 (2%), KCl (25 mM), glutamine (1 mM), penicillin (100 units/ml) and streptomycin (100 ug/ml). Cells were plated onto poly-D-lysine (50 μg/ml)-coated coverslips and maintained at 37°C in a humidified environment containing 5% CO_2_ for 72 hours. The maximal neurite length in each YFP-positive CGN was measured with Image Pro plus software.

For cerebellar microexplant culture, the tissues were chopped to small pieces which were passed through a 308 μm nylon mesh. The resulting tissue chunks were collected by sedimentation and resuspended in serum-free BME supplemented with hormones as previously described^32^. Cerebellar tissues were plated in coverslips that were double-coated with poly-D-lysine (20 μg/ml in sterile water overnight at room temperature) and laminin (20 μg/ml in PBS for 3 hours at room temperature). After allowing for attachment for 1–2 hours, an appropriate volume of medium (DMEM/F12 medium with 2 mM glutamine, 0.1 mg/ml BSA, 2.0% N2, 100 units/ml penicillin and 100 μg/ml streptomycin)^33^ was added. Explants were cultured for 36 hours, and the rate of CGN migration was measured by the method described previously with some modifications^34^. Briefly, we delimited concentric areas of 0–100, 100–200 or 200–400 μm width from the explant border. The intensity of YFP fluorescence in each area was measured using the Image-Pro Plus software and then expressed as a percentage of YFP intensity of the whole culture.

### Immunoprecipitation and immunobloting

Immunoprecipitation of FAK was performed as previously described^35^. Briefly, an aliquot of cell lysate containing 200 μg of proteins was pre-cleared with Protein A/G PLUS-Agarose. Protein A/G PLUS-Agarose was incubated with anti-FAK antibody for 2 hours at 4°C. Then the pre-cleared protein was treated with Protein A/G PLUS-Agarose/antibody complex at 4°C overnight. Immunoprecipitates were collected by centrifugation at 3,000 g for 1 minute. After washing five times with 0.01 M PBS, the pellets were resuspended in 20 μl 2 × sodium dodecyl sulfate sample buffer. Then the protein sample was analyzed with SDS-PAGE and immunobolting as previously described^31^.

### GST pull-down assay

Bacterial cells were lysed using the following buffer: 20 mmol/L Tris-Cl, 2 mmol/L EDTA, 150 mmol/L NaCl, 0.5% NP40, pH 7.5.The bacterial lysate containing GST-PACT fusion protein was incubated with glutathione-Sepharose 4B beads at 4°C overnight. The beads were washed and then incubated with COS7 cell lysate for 6 hours. After washing, the bound proteins were denatured from the beads and subjected to SDS-PAGE^36^.

### Immunohistochemistry

For immunohistochemical analysis, animals were anesthetized by intraperitoneal injection (IP) of chloral hydrate (500 mg/kg) and perfused with 10 ml of saline, followed by 100 ml of 4% paraformaldehyde in 0.1 M phosphate buffer (PB, pH 7.2)^37^. Then brain tissues were removed and post-fixed in the same fixative overnight then transferred to 30% sucrose for an additional 24 hours and the cerebella were sectioned with a sliding microtome (Microm Laborgerate GmbH, Germany) at the thickness of 25 μm.

The procedure for immunohistochemistry has been previously described^38^. Briefly, cerebellum free-floating sections were incubated in 0.01 M PBS containing 3% H_2_O_2_ and 40% methanol for 30 minutes at RT and then treated with 0.5% Triton X-100 (Sigma-Aldrich Co. LLC., St. Louis, MO) in PBS for 5 minutes. The sections were washed with 0.01 M PBS three times and blocked with 1% BSA and 0.05% Triton X-100 for 2 hours at RT and then incubated with primary antibody overnight at 4°C, followed by an incubation with secondary antibody for 2 hours at RT. After three washes in PBS, sections were incubated with Streptavidin, HRP-Conjugated (Calbiochem, Darmstadt, Germany) solutions for 2 hours and then developed in PBS containing 0.05% DAB and 0.003% H_2_O_2_.

### Immunofluorescence

Fixed cerebellum sections, microexplants or cultured cells were treated with 0.5% Triton X-100 in PBS for 5 minutes, incubated with blocking solution (1% BSA with 0.05% Triton X-100) for 30 minutes, and then treated with primary antibodies diluted in blocking solution for 1 hour. Cerebellum sections were blocked for 2 hours and then treated with primary antibodies diluted in blocking solution overnight. Sections were then washed with PBS and incubated with the secondary antibody and/or with Alexa Fluor® 555 phalloidin for 1 hour. After three washes with PBS, the sections were stained with DAPI. The immunofluorescent images were captured under Zeiss LSM510 meta confocal microscope (Carl Zeiss MicroImaging Inc., Germany).

### Statistical analysis

Statistical analyses were performed with PRISM software. Comparisons between two groups were evaluated by the Student's t test. Comparisons among multiple groups were performed with analysis of variance (ANOVA), followed by a two-tailed, unpaired Student's t test. Differences were considered significant when p < 0.05.

## Author Contributions

Y.Y., Y.M., J.L. and Z.J.K. wrote the main manuscript text; Y.Y., Y.M., H.D., Z.F., Y.T. and C.Z. conducted experiments; Y.Y. and Y.M. prepared figures. All authors reviewed the manuscript.

## Supplementary Material

Supplementary Information Supplementary information
