# Supplementary material for: PACT/RAX Regulates the Migration of Cerebellar Granule Neurons in the Developing Cerebellum
Source: Sci Rep. 2015 Jan 22;5:7961. doi: 10.1038/srep07961 (PMC4302322; doi:10.1038/srep07961)
Supplement: Supplementary Information [file srep07961-s1.pdf]

Supplementary information:

PACT/RAX Regulates the Migration of Cerebellar Granule Neurons in the Developing Cerebellum

Yue Yong[1,3*], Ya Meng[1,3*], Hanqing Ding[3], Zhiqin Fan[1], YifenTang[1], Chenghua Zhou[1,3], Jia Luo[2], Zun-Ji Ke[1,3]

1. Department of Biochemistry, Shanghai University of Traditional Chinese Medicine, 1200 Cailun Road, Shanghai 201203, China
2. Department of Molecular and Biochemical Pharmacology, University of KentuckyCollege of Medicine, Lexington, Kentucky40536, USA.
3. Key Laboratory of Nutrition and Metabolism, Institute for Nutritional Sciences, Shanghai Institutes for Biological Sciences, Chinese Academy of Sciences, Graduate School of the Chinese Academy of Sciences, Shanghai 200031, China

*Both authors contributed equally to this work.

Address correspondence to:

Zun-Ji Ke, e-mail: kezunji@shutcm.edu.cn; Tel: 86-021-51323112; Fax: 86-021-51322146; Department of Biochemistry, Shanghai University of Traditional Chinese Medicine, 1200 Cailun Road, Shanghai 201203, China.

Jia Luo, e-mail: jialuo888@uky.edu; Tel: 859-323-3036; Fax: 859-257-0199; Department of Molecular and Biochemical Pharmacology, University of Kentucky College of Medicine, 1095 Veterans Drive, Lexington, Kentucky 40536, U.S.A.

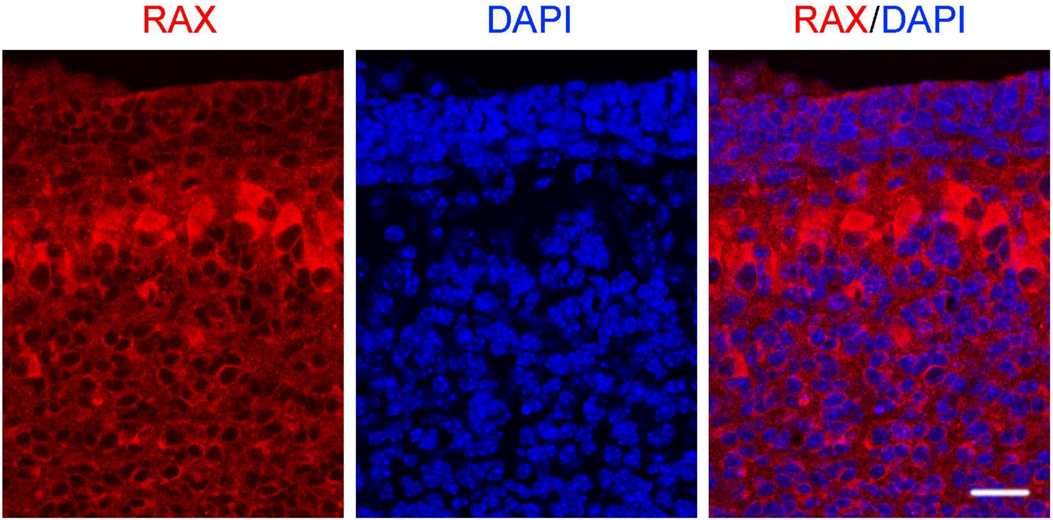

Suppl. Fig. 1. Confocal micrographs of the coronal section from PD4 mouse cerebellum labeled with RAX antibody (red) and DAPI (blue). Scale bar = 25 μm.

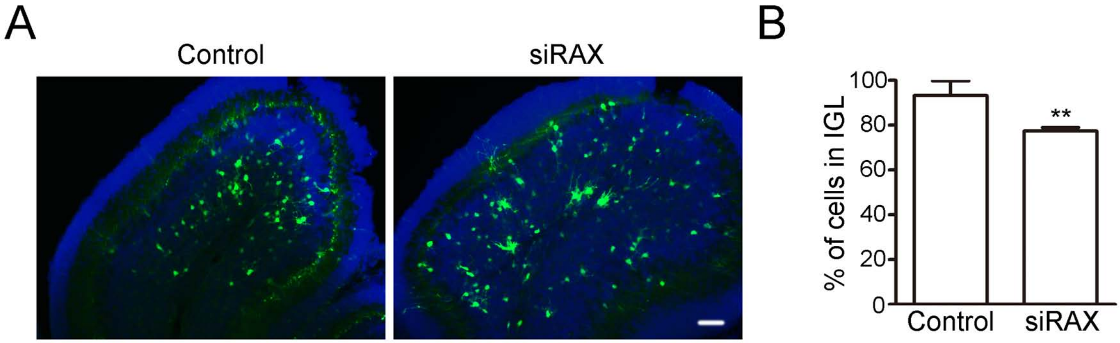

Suppl. Fig. 2. Effect of RAX knockdown on the migration of cerebellar granule neurons at 7 days post electroporation.
(A) Immunofluorescent images showing CGNs in control and siRAX treated groups at 7 days post electroporation. Scale bar = 50 μm.
(B) The percentage of cells that migrated into IGL was calculated at 7 days post the electroporation. Each data point was mean ± s.d. ($n \geq 5$), **$p<0.01$.

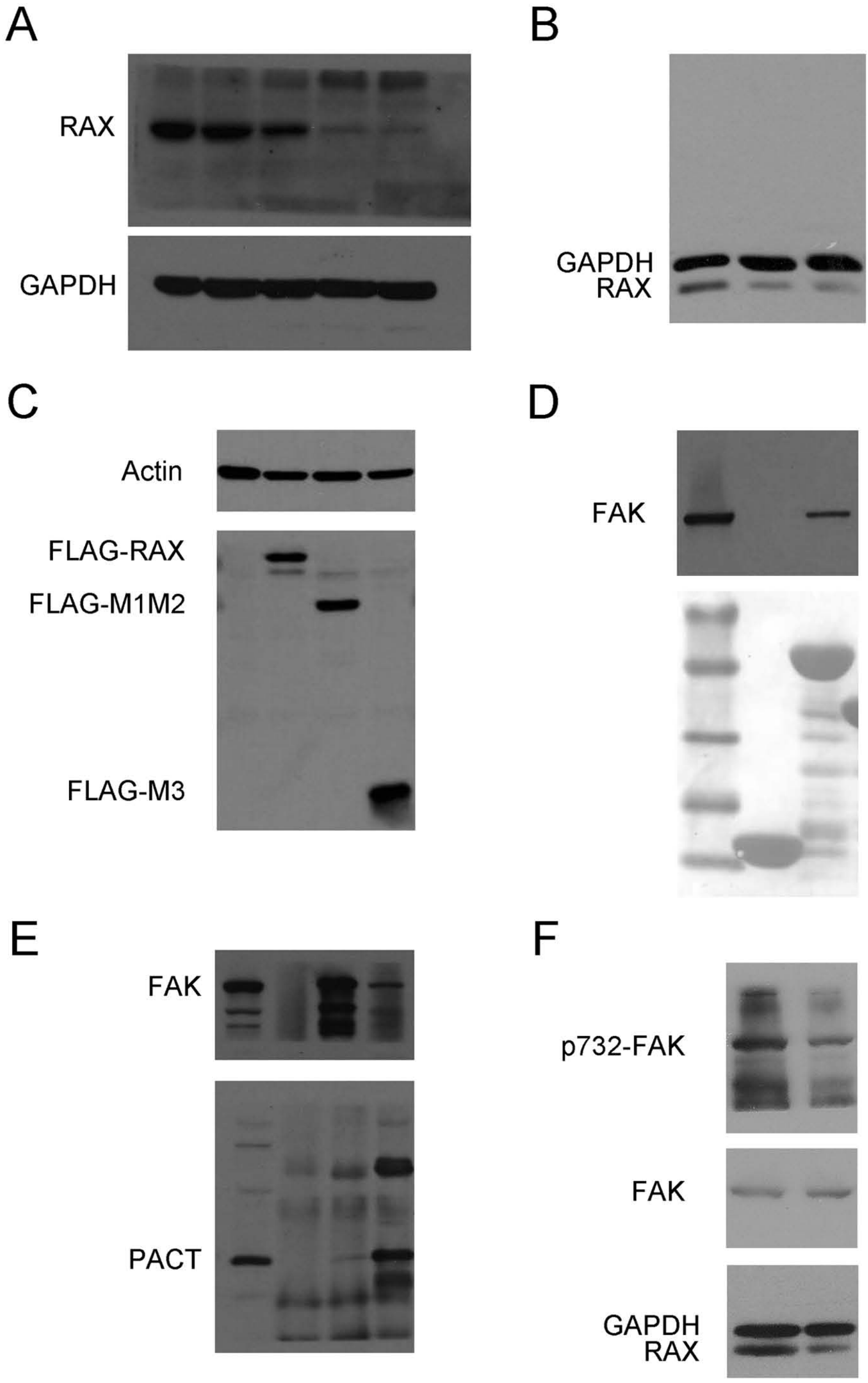

Suppl. Fig. 3. Full-length immunoblots of data shown in Figure 1A, 2A, 5B, 7A, 7B and 7C.
